# The Mechanism Underlying the Protective Effects of Tannic Acid Against Isoproterenol-Induced Myocardial Fibrosis in Mice

**DOI:** 10.3389/fphar.2020.00716

**Published:** 2020-05-15

**Authors:** Donglai Ma, Bin Zheng, Huiru Du, Xue Han, Xuan Zhang, Jianping Zhang, Yonggang Gao, Shijiang Sun, Li Chu

**Affiliations:** ^1^School of Pharmacy, Hebei University of Chinese Medicine, Shijiazhuang, China; ^2^Collaborative Innovation Center of Integrative Reproductive Disorders, Hebei University of Chinese Medicine, Shijiazhuang, China; ^3^Hebei Key Laboratory of Integrative Medicine on Liver-Kidney Patterns Hebei University of Chinese Medicine, Shijiazhuang, China; ^4^Department of Pharmaceutical Engineering, Hebei Chemical and Pharmaceutical College, Shijiazhuang, China; ^5^School of Basic Medicine, Hebei University of Chinese Medicine, Shijiazhuang, China; ^6^Hebei Province Hospital of Chinese Medicine, Affiliated Hospital, Hebei University of Chinese Medicine, Shijiazhuang, China

**Keywords:** tannic acid, cardioprotection, isoproterenol, toll-like receptor 4, nuclear factor κB, myocardial fibrosis

## Abstract

Tannic acid (TA) belongs to a class of complex water-soluble polyphenolic derivatives that show anticarcinogenic, antiinflammatory, antioxidant, and scavenging activities. Here, we investigate the protective effects of TA against isoproterenol (ISO)-induced myocardial fibrosis (MF) in mice. Mice received TA and ISO dosing and were sacrificed 48 h later. The activities of creatine kinase (CK), creatine kinase-MB (CK-MB), lactate dehydrogenase (LDH), and mitochondria enzymes were measured. Cardiac histopathology was done using H&E, Sirius red, and Masson’s Trichrome staining. Immunohistochemical staining was applied to indicate changes in B-cell lymphoma-2 (Bcl-2), Bcl-2-associated X protein (Bax), and basic fibroblast growth factor (bFGF) protein expressions in cardiac tissue. RT-PCR was used to measure the expression of atrial and brain natriuretic peptides (ANP and BNP, respectively), c-fos, and c-jun. Western blotting was used to measure the expression of nuclear factor-κB (NF-κB) p65, phosphorylated NF-κB p65), toll-like receptor 4 (TLR4), p38, phosphorylated p38, Bax, Bcl-2, and caspase-3. Compared to the ISO group, the TA group had reduced levels of TLR4, p38, p-p38, NF-κB (p65), p-NF-κB (p-p65), caspase-3, Bax, and Bcl-2, as well as CK, CK-MB, and LDH. These results indicate that TA protects against ISO-induced MF, possibly through its ability to suppress the TLR4-mediated NF-κB signaling pathway.

## Introduction

Despite increasing mean lifespan and living standards, cardiovascular disease (CVD) remains a major cause of death (Oka et al., 2014). Myocardial fibrosis (MF) is an important pathological change that eventually leads to heart failure. MF typically results from the continuous progression of CVD (e.g., myocardial infarction and hypertension) (Wu et al., 2009; Duygu et al., 2013). MF is a key pathological feature of myocardial remodeling and an important cause of CVD formation. MF manifests mainly as excessive collagen fiber deposition in cardiac muscle tissue, a significant increase in collagen content, an imbalance in the proportion of collagen types (increased proportions of I/III), and disorganized collagen arrangements. However, the mechanism underlying MF remains unclear (Elliott et al., 2000).

Excessive activation of the β-adrenergic receptor is an important factor contributing to MF (Ayala et al., 2012). Excessive activation of the β-adrenergic receptoris characterized by the normal activity of important myocardial neurohumoral factors, but long-term β-receptor activation can lead to myocardial interstitial cell prolife proportion, fibrous tissue hyperplasia, and excess amounts of collagen and cellulose (Boluyt et al., 1995). It has been shown that continuous use of the β-adrenergic receptor agonist isoproterenol (ISO) can induce MF in rats (Borges et al., 2003; Zhang et al., 2016a; Zhang et al., 2016b).

Toll-like receptors (TLRs) provide a link between the immune system and the development of CVD (Avlas et al., 2011). Among TLRs, TLR4 is associated with human CVDs and is an important receptor for mediating myocardial inflammatory signals (Yin et al., 2015). Activation of TLR4 can increase p38 MAPK and nuclear factor κB (NF-κB) (p65) representations, which in turn lead to the expression of inflammatory factors. NF-κB (p65) activities also play a role in the regulation of TLR4 (Freund et al., 2005; Eguchi and Manabe, 2014).

**Figure 1:**
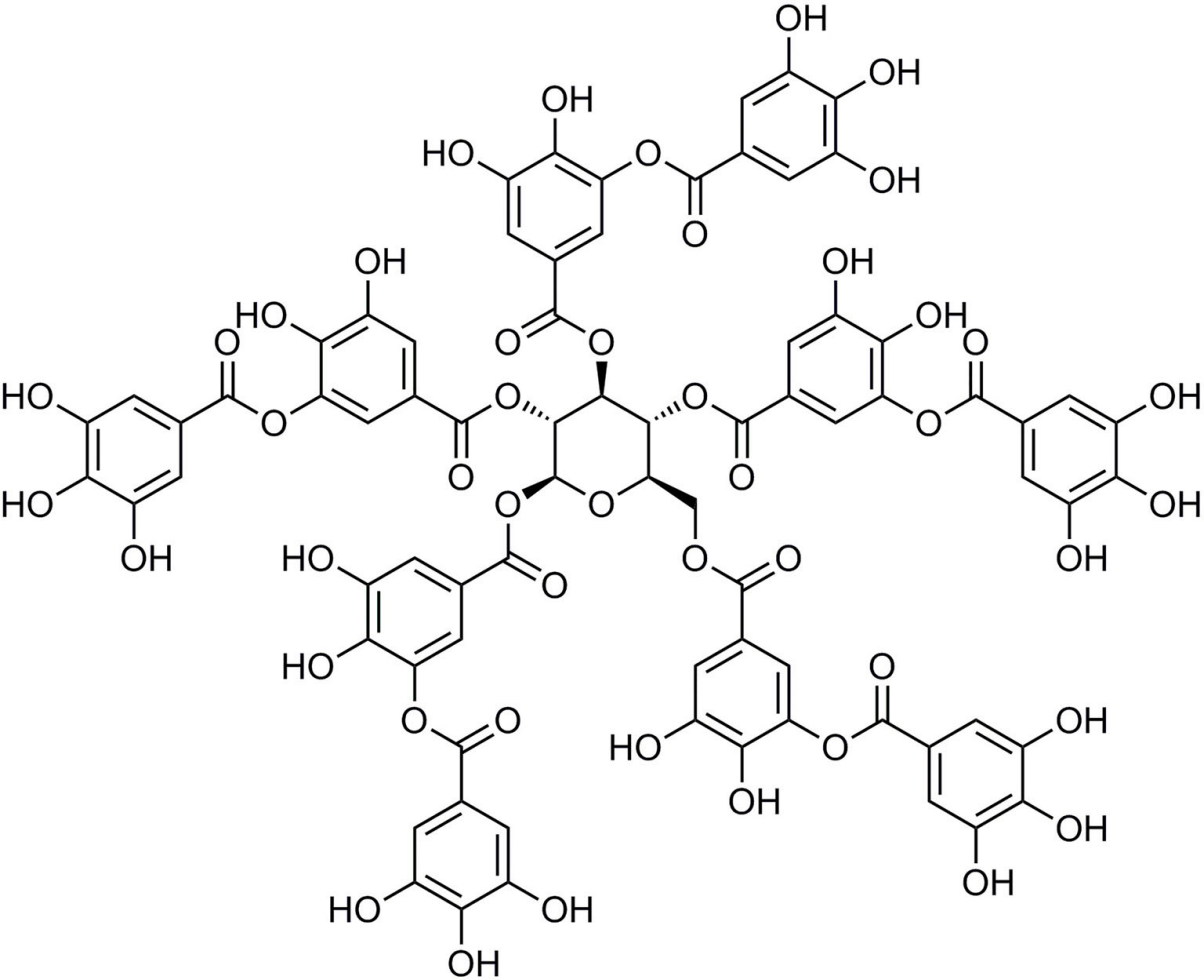
Tannic acid (TA) structure.

Tannic acid (TA; **Figure 1**) is a class of more complex plant polyphenol derivatives (Haslam et al., 1989). It is abundant in cereals, fruits, herbs, tea, and red wine (Henis et al., 1964; Thiansilakul et al., 2012). TA’s chemical structure contains glucose linked to a 3–5 gallic acid *via* an ester bond (Yugarani et al., 1993). Previous studies have suggested that TA possessesanti-lipogenic, anticarcinogenic, antiinflammatory, antioxidant, and scavenging activities (Gali et al., 1992; Chu et al., 2016). TA has some potent pharmacological effects, including myocardial protection (Hu et al., 2015; Zhang et al., 2017a; Zhang et al., 2017b), a decrease in L-type Ca^2+^ currents in isolated mice ventricular myocytes (Zhu et al., 2016), and a vasodilatory effect *via* activation of K^+^ channels expressed in HEK293 cells (Chu et al., 2015; Zhang et al., 2016a). However, the relevance of the protective results of TA on chronic MF is not well-defined.

To investigate whether TA has inhibitory effects on ISO-induced MF, we studied inflammation and apoptosis-associated MF mediators, such as TLR4, p38, p-p38, NF-κB (p65), p-NF-κB (p65), B-cell lymphoma-2 (Bcl-2), and Bcl-2-associated protein (Bax). It has been reported that the β-adrenergic receptor blocker of propranolol (Pro) provides significant protection against ISO-induced MF (Zheng et al., 2007). Therefore, Pro was applied as a positive control drug.

## Materials and Methods

### Animals

Male Kunming mice (KM, 20–22 g) were purchased from Hebei Medical University (Shijiazhuang, China). Animals were free to consume food and water in a standard laboratory environment (12/12 h day and night cycle, 25°C ± 1°C, 55% ± 10% humidity) and subcage-adapted for 7 days. All experiments conformed to the Guide for the Care and Use of Laboratory Animals published by the US National Institutes of Health (NIH Publication, 8th Edition, 2011). This study was carried out following the recommendations of the Declaration of Helsinki. The protocol was approved by the Hebei University of Chinese Medicine Committee on Animal Care.

### Reagents

TA was purchased from Sigma-Aldrich (Shanghai, China) (**Figure 1**). ISO was obtained from Amylet Scientific (Michigan, USA). Propranolol was purchased from Afar Sally Chemical Co. (Tianjin, China).

### Preexperiment

Methods from other literature reports, including ISO dose, modeling cycle, and safety issues, were used to investigate the mouse MF model. Twelve Kun Ming (KM) mice were administered subcutaneously with ISO at 10 mg/kg/day and mice were sacrificed 14 days after continuous administration. We detected obvious pathological MF changes that were consistent with those from other reports (Wu et al., 2009). The mouse MF model was also shown to be successful. The modeling method and ISO dose were shown to be safe and reliable in mice.

### Experimental Design

The animals were randomly (random number table method) divided into groups. Fifty healthy KM mice were randomized into five groups: (1) control group (CONT, n = 10), subcutaneously administered normal saline (10 mg/kg/day) subcutaneous and intraperitoneal; (2) ISO group (n = 10), subcutaneously administered ISO (10 mg/kg/day) plus normal saline (10mg/kg/day); (3) Pro group (n = 10), subcutaneously administered ISO (10 mg/kg/day) plus (Pro 40 mg/kg/day); (4) low-dose TA group (L-TA, n = 10), subcutaneously administered ISO (10 mg/kg/day) plus TA (20 mg/kg/day); and (5) high-dose TA group (H-TA, n = 10), subcutaneously administered ISO (10 mg/kg/day) plus TA (40 mg/kg/day).

Body weight gains and feed efficiencies were calculated. All mice were sacrificed after 14 days. Blood was gathered retro-orbitally at room temperature, then centrifuged at 3000 rpm for 30 min and preserved at −20°C. The hearts were quickly separated, weighed, and preserved at −80°C or fixed with 10% paraformaldehyde.

### ELISA Assay

The isolated serum sample was used for the diagnosis of labeled enzymes. Total serum CK, CK-MB, and LDH were detected using the velocity method at 37°C using the CK, CK-MB, and LDH kit (Jiancheng Biological Engineering Institute, China), which is based on an enzyme-coupled reaction.

### Detection of Mitochondrial Enzyme Activities

A well-ground 10% homogenate of heart tissue was centrifuged at 3,000 rev/min for 10–15 min at low temperature to prepare a supernatant for the determination of mitochondrial enzyme activities. The supernatant at 100,000 g were centrifuged for 1 h and the precipitate is mitochondria. The separated mitochondria were made into a suspension with 4°C homogenized medium, and the mitochondria were broken three to five times in an ice-water mixture using an ultrasonic generator 400 mA, 5 s/times, and a gap of 10 s. After the coomassie brilliant blue method was used to determine the protein content, aliquots were stored in a refrigerator at −80°C for future use. Na^+^, K^+^-ATPase and Ca^2+^, Mg^2+^-ATPase activities of myocardial mitochondria were measured according to the operating instructions of the ATPase kit (Jiancheng Biological Engineering Institute, China).

### H&E, Sirius Red, and Masson’s Trichromestaining

Mice hearts fixed in 4% paraformaldehyde solution were taken from the same location. The hearts were then paraffin-embedded after routine treatment and sliced into 4-μm sections. The sections were stained with H&E, Sirius red, and Masson’s trichrome staining. After staining was complete, collagen fibers were surveyed under a light microscope. According to the semiquantitative scoring system for MF, the degree of MF in each group was scored. A higher score indicates a more severe degree of fibrosis.

### Immunohistochemistry

The paraffin specimens were sectioned for immunohistochemistry. The sections were boiled in 10 mM sodium citrate buffer for 20 min, then cooled to room temperature. Following antigen retrieval, sections were peroxidase blocked in 3% H_2_O_2_ for 10 min and blocked with 10% normal goat serum at 37°C for 15 min. After incubation with the primary antibody, the slides were incubated with diluted rabbit polyclonal antibodies to Bax (AB32503, Abcam, Cambridge, UK; 1:65), Bcl-2 (AB32124, Abcam, Cambridge, UK; 1:70), or bFGF (AB4222, Abcam, Cambridge, UK; 1:60) at 4°C overnight. The streptavidin/peroxidase complex was applied to slide sections and incubated at 37°C for 15 min and 3,3'-diaminobenzidine hydrochloride was added to the tissue portions. The sections were counterstained with hematoxylin and sealed with neutral gum. Five pictures (×400) per heart were recorded randomly through a digital microscope (Olympus) and quantified with LAS 4.2 software.

### Reverse Transcription PCR

The Trizol reagent was used to extract total myocardial RNA. For the total RNA, the absorbance was measured at 260/280 nm using a microplate reader. Total RNA (2 μg) was reverse transcribed into cDNA using an reverse transcription PCR (RT-PCR) synthesis kit (TaKaRa Bio Inc., Dalian, China). The sequences of primers were as follows: ANP (sense 5′-GGCTTCTTCCTCGTCTTGG-3′; antisense 5′-ATCTGTGTTGGACACCGCA-3′); BNP (sense 5′- CGGTCTCAAGGCAGCAC-3′; antisense 5′-GTTACAGCCCAAACGACT-3′); c-fos (sense 5′-ACGGACTCCCCACCCAG-3′; antisense 5′-CGTTCCCTTCGGATTCT-3′); c-jun (sense 5′-GCCCCTGTCCCCTATCG-3′; antisense 5′-TCCAGCTTCCTTTTCCG-3′); and β-actin (sense 5′-GAGAGGGAAATCGTGCGTGACA-3′; antisense 5′-ACCCAAGAAGGAAGGCTGGAAA-3′) served as the internal control.

The RT parameters were: (1) 30°C for 10 min; (2) 42°C for 30 min; (3) 5°C for 5 min. RT-PCR was done according to the manufacturer’s instructions. The reaction consisted of: (1) 94°C for 5 min; (2) 94°C for 30 s; (3) 72°C for 10 min; 35 cycles. The PCR products were analyzed by 1.5% (w/v) agarose gel electrophoresis. The GL2200PROgel imaging system was applied to scan the gray scale of the specific PCR products amplified by each sample. The relative transcript levels of ANP, BNP, c-fos, and c-jun were normalized to β-actin.

### Western Blotting

The RIPA lysate (1 mmol/L PMSF) was added to each group’s hearts in an ice bath with stirring for 0.5 h. The supernatant was centrifuged for 0.5 h, and protein concentrations were determined using the bicinchoninic acid (BCA) method (BCA kit, SangonBiotech, Shanghai, China). The proteins were separated on SDS-PAGE and transferred to nitrocellulose membranes. The membranes were blocked with 5% skim milk powder at 24°C for 2 h and then incubated with primary antibody rabbit anti-rat TLR4 (1:1,000 dilution), p38 (1:1,000 dilution), p-p38 (1:500 dilution), NF-κB (p65) (1:3,000 dilution), p-NF-κB (p65) (1:1,000 dilution), Bax (1:1,000 dilution), Bcl-2 (1:1,000 dilution), caspase-3 (1:1,000 dilution), and mouse antibody β-actin at a 1:1,000 dilution at 4°C for 10 h. After rewarming, the membrane was blocked with a secondary antibody for 90 min. The membrane was washed, subjected to chemiluminescence, developed, and fixed. Images were acquired using a Fluor Chem Q multifunctional imaging and quantitative analysis system. With β-actin as an internal reference, the proportion of absorbance value between the target protein and reference absorbance value represented the relative expression content of the target protein.

### Statistical Methods

The data are expressed as the mean ± standard error of the mean, and the differences between the groups were quantified by analysis of variance (ANOVA) or Dunnett's T-test. P-values <0.05 were considered to be statistically signiﬁcant. All statistical analyses were done using the Statistical Packagefor Social Science (SPSS, Chicago, IL),Version 24.0.

## Results

### Effects of TA on Levels of CK, CK-MB, and LDH

**Figure 2:**
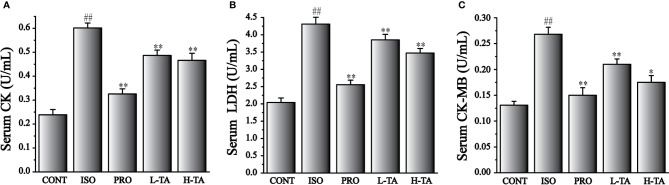
The effects of TA on serum levels of creatine kinase (CK) **(A)**, lactate dehydrogenase (LDH) **(B)**, and CK-MB **(C)**. Data represent mean ± SEM. Compared to the CONT group (^##^P < 0.01). Compared to the isoproterenol (ISO) group (*P < 0.05 and **P < 0.01).

The CK, CK-MB, and LDH levels in serum were tested in accordance with the kit method. In **Figure 2**, ISO-induced significant increases in CK, CK-MB, and LDH compared with CONT group. Compared with the ISO group, CK, CK-MB, and LDH serum levels in per group had significantly decreased (P<0.05 and P<0.01). In MF mice, H-TA group was better able to lower serum CK, CK-MB, and LDH levels than L-TA group.

### Effects of TA on Mitochondrial Enzyme Activities

**Figure 3:**
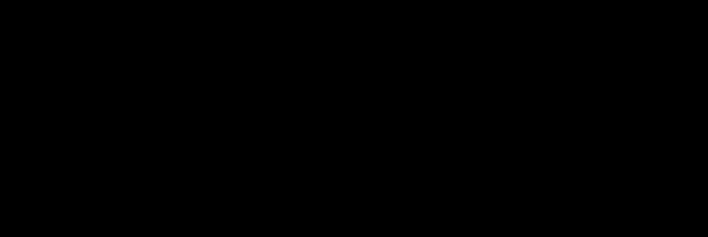
The effects of tannic acid (TA) on mitochondrial enzyme activities. Data represent mean ± SEM. Compared to the CONT group (^##^P < 0.01). Compared to the isoproterenol (ISO) group (*P < 0.05 and **P < 0.01).

Mitochondrial enzyme activities in each group is shown in **Figure 3**. ISO-induced significant reduces Na^+^, K^+^-ATPase and Ca^2+^, Mg^2+^-ATPase activities of myocardial mitochondria compared with CONT group. Compared with the ISO group, Na^+^, K^+^-ATPase and Ca^2+^, Mg^2+^-ATPase activities in per group had significantly increased (P<0.05 and P<0.01). In MF mice, H-TA group was better able to higher Na^+^, K^+^-ATPase and Ca^2+^, Mg^2+^-ATPase activities than L-TA group.

### Myocardium Pathology

#### H&E Staining

**Figure 4:**
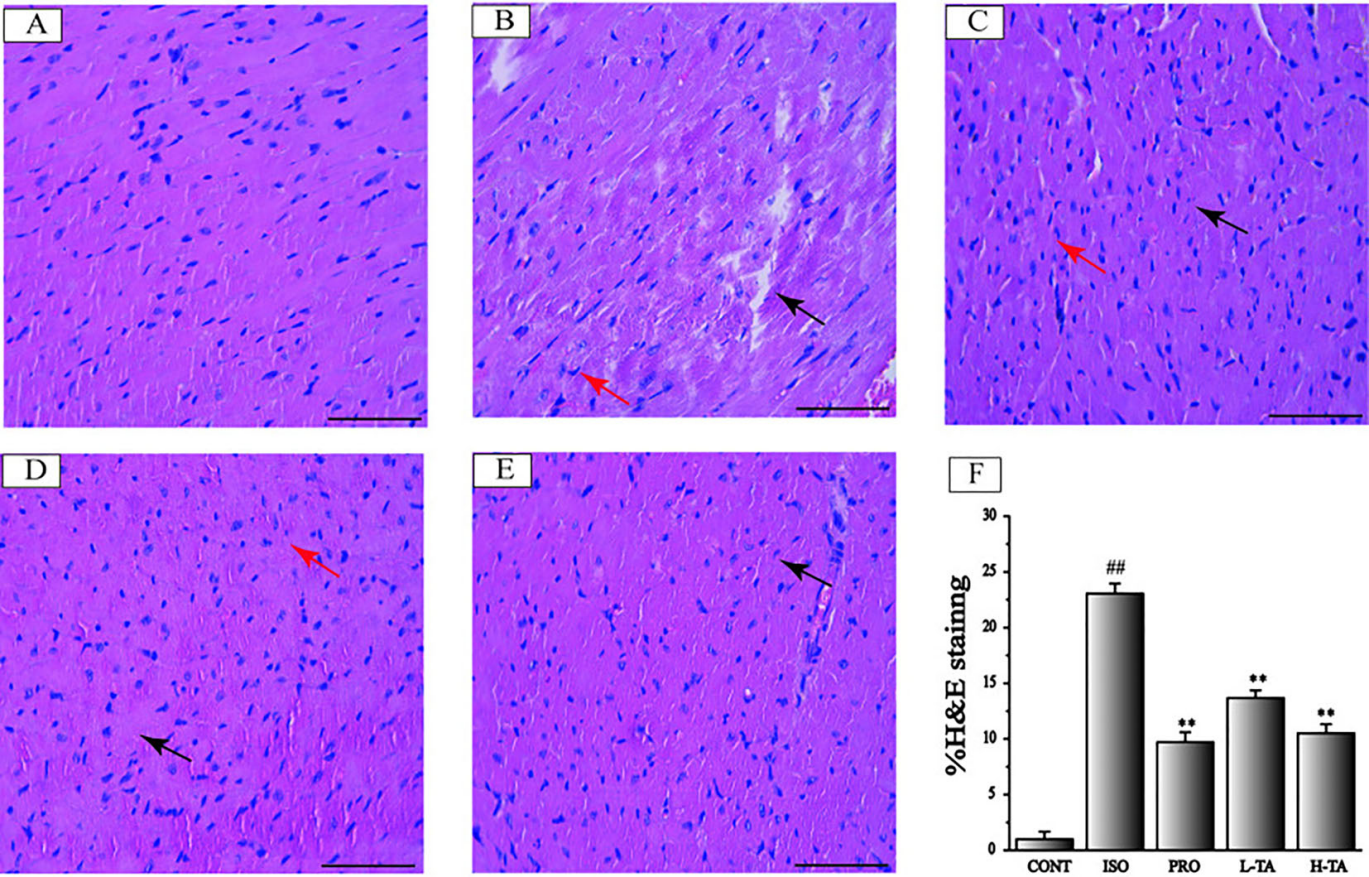
The effects of tannic acid (TA) on cardiac ﬁbrosis as surveyed by H&E staining. The staining is shown for the CONT **(A)**, isoproterenol (ISO) **(B)**, PRO **(C)**, low-dose TA (L-TA) **(D)**, and high-dose TA (H-TA) **(E)** groups (H&E, 400×; bar 50 μm). Pay attention note the cardiomyocytes’ derangement distribution (arrows) as revealed with H&E staining. **(F)** Quantification of the H&E staining expressed as the proportion of the area positive for collagen fibers. Data represent mean ± SEM. Compared to the CONT group (^##^P < 0.01). Compared to ISO group (**P < 0.01).

H&E staining of myocardial tissue in each group is shown in **Figure 4**. The proportion of the positive area of collagen fibers was calculated using Image-Pro Plus 5.0. In the CONT group, MF was arranged in neat rows showing myocardial cells without atrophy or hypertrophy, indicating normal myocardial structure. The fibers in the ISO group of MF were disrupted and disordered, and the myocardial interstitial collagen fibers increased dramatically (P<0.01). Contrasted with the ISO group, the TA groups had dramatically decreased myocardial interstitial collagen fibers (P<0.01), especially in the H-TA group in which myocardial interstitial fibers occasionally showed a small amount of collagen fibrosis and inflammatory cell infilt proportion.

#### Sirius Red Staining

**Figure 5:**
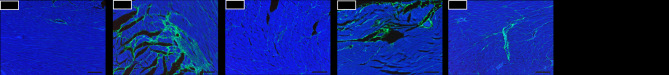
The effects of tannic acid (TA) on cardiac ﬁbrosis as surveyed by Sirius red staining. The staining is shown for the CONT **(A)**, isoproterenol (ISO) **(B)**, PRO **(C)**, low-dose TA (L-TA) **(D)**, and high-dose TA (H-TA) **(E)** groups (200×; bar 100 μm). **(F)** Quantification of Sirius red staining expressed as the proportion of the positive area for collagen fibers. Data represent mean ± SEM. Compared to the CONT group (^##^P < 0.01). Compared to ISO group (*P < 0.05 and**P < 0.01).

Sirius red staining has been reported as the most appropriate stain in the evidentiation of MF (Cowling et al., 2019). Sirius red stained heart sections from the control group were observed under the microscope (**Figure 5**). Progressive interstitial fibrosis was observed in the ISO group. Versus the ISO group however, TA treatment significantly attenuated MF. Furthermore, myonecrosis lead to less edema, fewer inflammatory cells, and a decreased degree of myocardial structural disorder in the mesenchyme. As a positive control, the ISO+PRO also significantly relieved MF. The effect of TA was slightly weaker than PRO. In conclusion, TA could alleviate ISO-induced MF.

#### Masson’s Trichrome Staining

**Figure 6:**
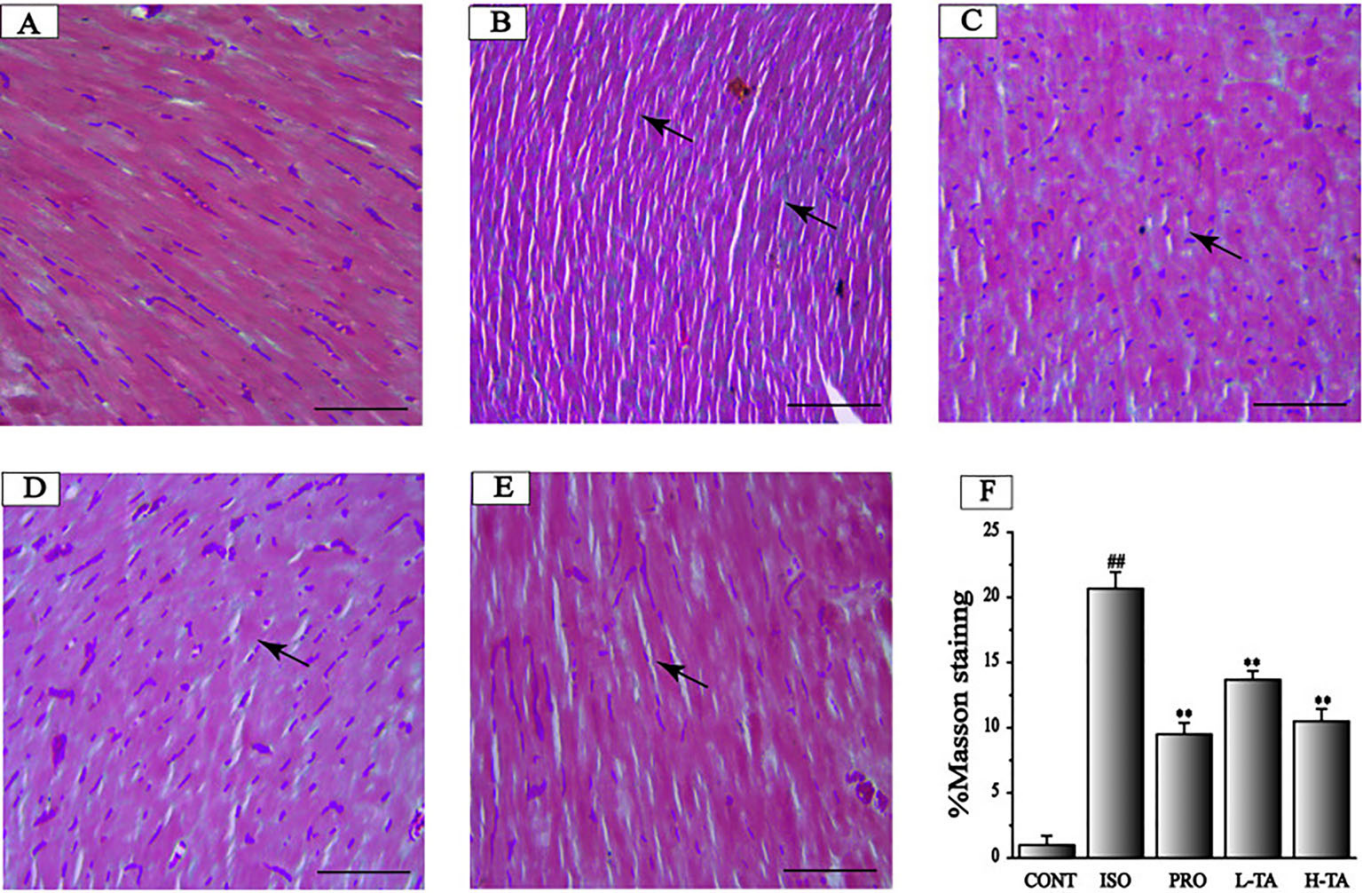
The effects of tannic acid (TA) on myocardial ﬁbrosis as surveyed by Masson's trichrome staining. The staining is shown for the CONT **(A)**, isoproterenol (ISO) **(B)**, PRO **(C)**, low-dose TA (L-TA) **(D)**, and high-dose TA (H-TA) **(E)** groups (Masson's trichrome staining, 400×; bar 50 μm). **(F)** Quantification of Masson's trichrome staining expressed as the proportion of the positive area for collagen fibers. Data represent mean ± SEM. Compared to the CONT group (^##^P < 0.01). Compared to the ISO group (**P < 0.01).

Masson's trichrome staining of myocardial tissue is shown in **Figure 6**. For the CONT group mice, ventricular myocardial and myocardial interstitial collagen fibers can be seen, with a small amount of deposition. Collagen deposition in the ISO group was more pronounced. In the TA group, collagen deposition has been alleviated. The proportion of the surveyed area that was positive for collagen fibers was measured using Image-Pro Plus 5.0. Relative to the CONT group, the myocardial collagen fibers in the ISO group were dramatically increased. Compared to the ISO group, each TA group had dramatically decreased myocardial interstitial collagen fibers (**Figure 6F**).

#### Results of TA on the Representation of Bax, Bcl-2, and bFGF

**Figure 7:**
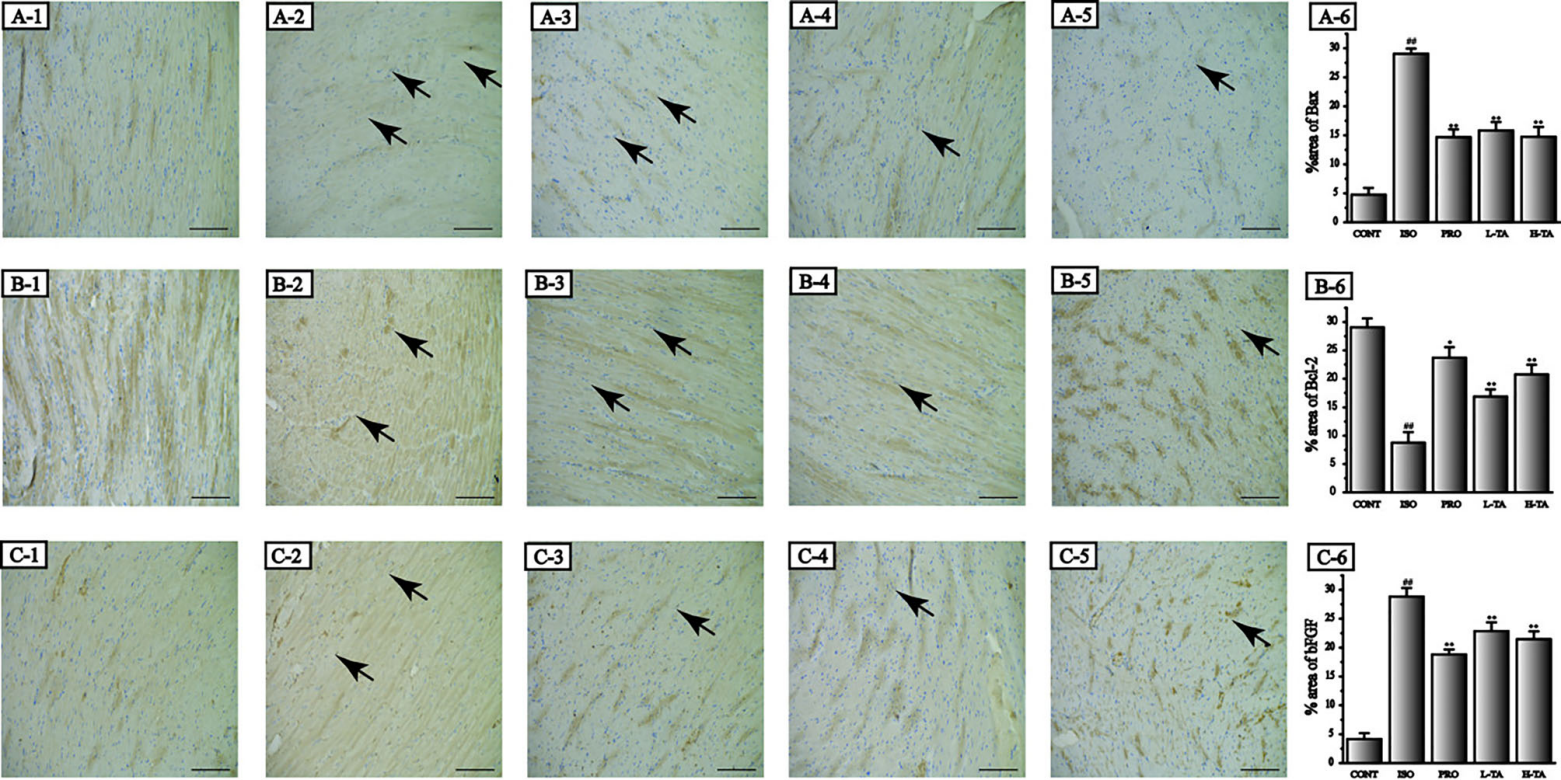
The effects of tannic acid (TA) treatment on Bax, Bcl-2, and basic fibroblast growth factor (bFGF) expression in heart observed after immunohistochemical dispose of. The morphological locations and percent areas of Bax (A1-6), Bcl-2 (B1-6), and bFGF (C1-6) expressions are shown (immunohistochemical, 400×; bar 50 μm). Positive expressions of Bax, Bcl-2, and bFGF are indicated by arrows. Data represent the mean ± SEM. Compared to the CONT group (^##^P < 0.01). Compared to the isoproterenol (ISO) group (*P < 0.05 and **P < 0.01).

Immunohistochemistry was applied to detect Bax, Bcl-2, and bFGF expression levels in each group (**Figure 7**). The staining results were analyzed using Image-Pro Plus 5.0 software. We found that, compared to the CONT group, positive staining of Bax and bFGF and parenchymal areas increased dramatically in the ISO group (P < 0.01), while Bcl-2 expression decreased. Compared to the ISO part, each TA group had a significant decrease in Bax, Bcl-2, and bFGF expressions in the myocardium (P < 0.01), while Bcl-2 expression increased.

### Effects of TA on the Expression of Bax/Bcl-2 and Caspase-3

**Figure 8:**
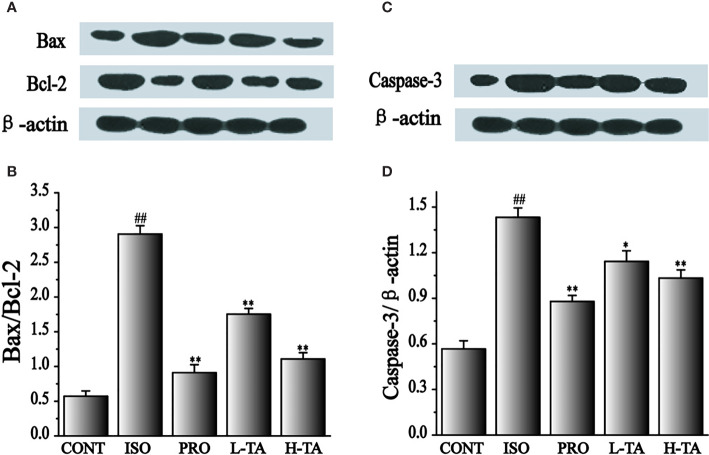
The effects of tannic acid (TA) treatment on Bax, Bcl-2, and caspase-3 expressions as shown by Western blotting (WB) analysis. The band intensities of Bax/Bcl-2 **(A, B)** and caspase-3 **(C, D)** were analyzed by normalization to β-actin. Data represent mean ± SEM. Compared to the CONT group (^##^P < 0.01). Compared to the isoproterenol (ISO) group (*P < 0.05 and **P < 0.01).

In the present study, the proportion of Bax/Bcl-2 and caspase-3 proteins was analyzed by western blotting. As shown in **Figure 8**, when compared with the CONT group, the proportion of Bax/Bcl-2 and caspase-3 proteins in the myocardial model of the ISO model group had increased dramatically (P < 0.01), indicating that ISO could induce expression of Bax/Bcl-2 and caspase-3 proteins in cardiomyocytes. Compared with the ISO group, the proportion of Bax/Bcl-2 and caspase-3 in the myocardium of mice were dramatically lower than in the TA group (P < 0.01), indicating that different TA doses can inhibit the expression of Bax/Bcl-2 and caspase-3 proteins in cardiomyocytes.

### Effects of TA on the Expression of ANP, BNP, c-fos, and c-jun

**Figure 9:**
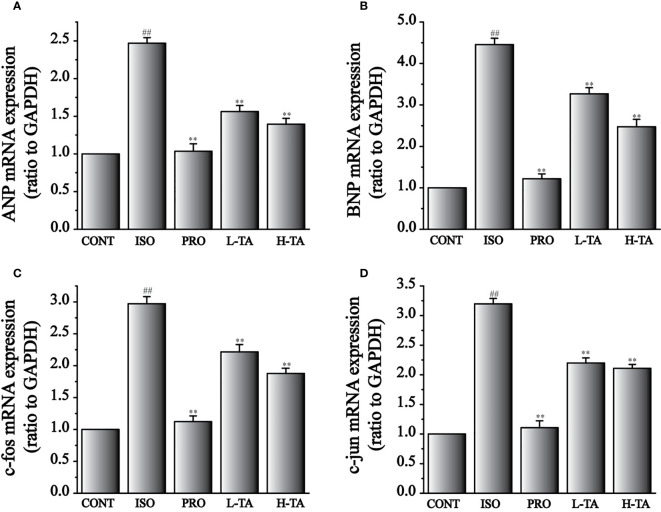
The effects of tannic acid (TA) treatment on atrial natriuretic peptide (ANP) **(A)**, brain natriuretic peptide (BNP) **(B)**, c-fos **(C)**, and c-jun **(D)** mRNA expressions, as shown by RT-PCR. Data represent mean ± SEM. Compared to the CONT group (^##^P < 0.01). Compared to the isoproterenol (ISO) group (**P < 0.01).

**Figure 10:**
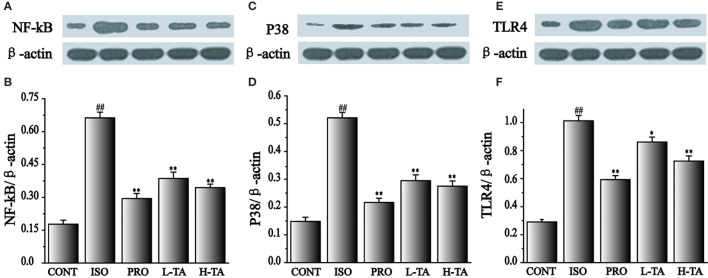
The effects of tannic acid (TA) treatment on nuclear factor-κB (NF-κB) (p65), p38, and toll-like receptor 4 (TLR4) expression, as shown by Western blotting (WB) analysis. Quantification of the NF-κB (p65) **(A**, **B)**, p38 **(C**, **D)** and TLR4 **(E**, **F)** protein expression normalization to the β-actin. The mean ± SEM are shown. Compared to the CONT group (^##^P < 0.01). Compared to the isoproterenol (ISO) group (*P < 0.05 and **P < 0.01).

The expression levels of ANP, BNP, c-fos, and c-jun mRNA were analyzed by RT-PCR (**Figure 10**). Compared to the CONT group, ANP and BNP expressions in the ISO group increased dramatically (P <0.01) (**Figures 9A, B**). When compared with the ISO group, mice myocardial ANP and BNP expressions dramatically decreased (P<0.01), indicating that TA could dramatically inhibit MF expression of ANP and BNP.

Compared with the CONT group, expressions of c-fos and c-jun mRNA increased dramatically in the ISO group (P < 0.01) (**Figures 9C, D**). Compared with the ISO group, c-fos and c-jun expressions in the myocardium dramatically decreased (P < 0.01), indicating that TA could dramatically inhibit MF expressing c-fos and c-jun mRNA.

### Effects of TA on the Expression of NF-κB (p65), p38, and TLR4

In this study, NF-κB (p65), p38, and TLR4 proteins were analyzed by WB. As shown in **Figure 10**, when compared with the CONT group, the NF-κB (p65), p38, and TLR4 proteins in the myocardial model of the ISO group had increased dramatically (P < 0.01), indicating that ISO could induce NF-κB (p65), p38, and TLR4 proteins in cardiomyocytes. Compared with the ISO group, the NF-κB (p65), p38, and TLR4 proteins in the myocardium were dramatically decreased in the TA group (P < 0.01), indicating that different TA doses can dramatically inhibit NF-κB (p65), p38, and TLR4 proteins in cardiomyocytes.

### Effects of TA on the Expression of Phosphorylated NF-κB (p65) and p38

**Figure 11:**
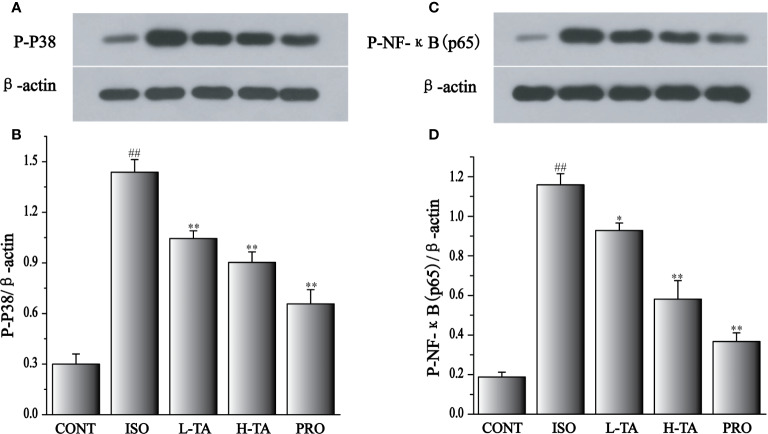
The effects of tannic acid (TA) treatment on phosphorylated nuclear factor-κB (NF-κB) (p65), and p38 expression, as shown by Western blotting (WB) analysis. Quantification of the phosphorylated p38 **(A, B)**, and NF-κB (p65) **(C, D)** protein expression normalization to the β-actin. The mean ± SEM are shown. Compared to the CONT group (^##^P < 0.01). Compared to the isoproterenol (ISO) group (*P < 0.05 and **P < 0.01).

As shown in **Figure 11**, when compared with the CONT group, the p-p65 and p-p38 proteins in the myocardial model of the ISO group had increased obviously (P < 0.01), indicating that ISO could induce p-p65 and p-p38 proteins in cardiomyocytes. Compared with the ISO group, the p-p65 and p-p38 proteins in the myocardium were dramatically decreased in the TA group (P < 0.01 and P < 0.05), indicating that different TA doses can dramatically inhibit phosphorylated NF-κB (p65) and p38 proteins in cardiomyocytes.

## Discussion

TA is commonly consumed in our daily life, such as in fruits, vegetables, wines, and tea. In the United States, on average, people consume 1 g of TA per day (Sanyal et al., 1997). Based on the formula proposed by Reagan-Shaw et al. (Reagan-Shaw et al., 2008), the doses (20 and 40 mg/kg/day) used in our experiment are close to people’s typical daily consumption. In recent years, research on TA has shown that its inhalation attenuates intermittent hypoxia (Sato et al., 1999; Liang et al., 2009) or ischemia/reperfusion (Zhao et al., 2010). Our results provide new experimental evidence for the MF of action of TA and might help to expand clinical treatments for CVD.

The results of in vivo experiments showed that the CK, CK-MB, and LDH of mice decreased after 2 weeks of intraperitoneal TA injections as well as the activities of myocardial mitochondrial enzyme were significantly increased. Myocardial tissue H&E, Sirius red, Masson’s trichrome staining and immunohistochemical results also showed that TA could dramatically improve myocardial tissue morphology, alleviate myocardial mitochondrial damage, reduce collagen deposition, and reverse MF.

ISO can lead to apoptosis of cardiomyocytes. Apoptosis is an important reflection of harmful cell stimuli, which is of great significance for maintaining body homeostasis (O’Neill et al., 2009). TRL4 is a member of the signal transduction family (Shyu et al., 2010). Upon stimulation with exogenous LPS or endogenous ligands, TRL4 binds to the adapter protein MyD88 and is then processed through one of two ways (Li et al., 2004; Liu et al., 2015): (1) signaling through the pathway that includes the Rel family transcription factor NF-κB (p65); or (2) through the P38 MAPK and jnk pathway, which activates transcription factors, such as transcriptional activator II, and participates in signal transduction, transcription activation, and regulation of target gene expression.

NF-κB (p65) can be activated by a variety of cardiac hypertrophy stimulators (Gupta et al., 2008). Expressions of its downstream embryonic genes (e.g., ANP and BNP) increased dramatically, leading to hypertrophy of the myocardial cells (Zhang et al., 2003). It has been reported that continuous ISO injections can increase p-38 protein expression (Li et al., 2017; Zhang et al., 2016b), which is an important mediator of ISO-induced cardiac fibrosis in the pathogenesis. P38 MAPK is covered in intracellular formation transfer. P38 MAPK responds to a wide range of extracellular stimuli and mediates cell growth, development, differentiation, and death (Qiu et al., 2001). For example, Yin et al. applied the specific p38 MAPK inhibitor, SB203580, to study the regulatory results of p38 MAPK on Bcl-2 and found that the decrease in Bcl-2 expression slowed down, indicating that p38 MAPK is covered in the regulation of Bcl-2 (De Chiara et al., 2006; Yin et al., 2006). Feidantsis and others also confirmed p38 MAPK involvement in proto-oncogene c-jun mRNA upregulation and protein expression (Feidantsis et al., 2012).

In this study, we found that the ANP, BNP, c-fos, and c-jun mRNA levels were lower in the TA treatment group than in the ISO group. In the ISO group, the expression of the apoptotic proteins had dramatically increased in the heart tissue of the mice. Compared to the ISO model group, the proportion of Bax/Bcl-2 and caspase-3 were dramatically decreased in the myocardium after TA group.

Recently, it has been suggested that TNF-α upregulates TLR4 expression by activating NF-κB (p65), leading to an upregulation of Bax/Bcl-2 and Caspase-3. These experimental results show that in the TA group, the apoptotic protein expressions [NF-κB (P65), TLR4, p38] had effectively resisted with ISO group. However, determining whether the NF-κB (P65)/TLR4 signaling pathway is involved in the protective effects of TA injection on cardiac hypertrophy will require further investigation.

## Conclusion

Overall, the present results indicate that TA exerts significant cardioprotective effects in mice with ISO-induced cardiac fibrosis. This protection might beachieved by suppressing the increases in NF-κB (P65), TLR4, p38 expression. Therefore, TA significantly ameliorated ISO-induced MF, possibly through its ability to suppress the TLR4-mediated NF-κB signaling pathway.

## Data Availability Statement

The raw data supporting the conclusions of this article will be made available by the authors, without undue reservation, to any qualified researcher.

## Ethics Statement

The animal care and experimental protocols were verified by the Hebei University of Chinese Medicine Committee on Animal Care, and then fulfilled according to the ethical standards laid down in the 1964 Declaration of Helsinki and its later amendments.

## Author Contributions

YG, SS, and LC conceived the experiments. HD and XH collected the samples. DM, BZ, and XH conducted the experiments. DM, JZ, and LC wrote the manuscript. DM and XZ analyzed the results. All authors read and approved the final manuscript.

## Funding

This work was supported by the Natural Science Fund of Education Department of Hebei Province (No. ZD2019006).

## Conflict of Interest

The authors declare that the research was conducted in the absence of any commercial or financial relationships that could be construed as a potential conflict of interest.
